# Molecular analysis of p21 and p27 genes in human pituitary adenomas.

**DOI:** 10.1038/bjc.1997.521

**Published:** 1997

**Authors:** H. Ikeda, T. Yoshimoto, N. Shida

**Affiliations:** Department of Neurosurgery, Tohoku University School of Medicine, Sendai, Japan.

## Abstract

**Images:**


British Journal of Cancer (1997) 76(9), 1119–1123
© 1997 Cancer Research Campaign

### Molecular analysis of *p21* and *p27* genes in human pituitary adenomas

**H Ikeda, T Yoshimoto and N Shida**

Department of Neurosurgery, Tohoku University School of Medicine, 1-1 Seiryo-machi, Aoba-ku, Sendai 980–77, Japan

**Summary** Pituitary tumours develop at a high frequency in *p27*-knockout mice and retinoblastoma gene-knockout mice, which suggests that cell cycle regulatory genes, such as cyclin-dependent kinase inhibitor genes, are involved in the tumorigenesis of pituitary adenoma. Analysis of *p21* and *p27* gene abnormalities in human pituitary adenoma was performed in 28 pituitary adenomas by polymerase chain reaction–single-strand conformational polymorphism. No point mutations were detected in these genes. As no abnormalities of the *p21* and *p27* genes were observed, and if these genes are indeed inactivated, it is likely to be via transcriptional or translational defects.

**Keywords:** cyclin-dependent kinase inhibitor; polymerase chain reaction–single-strand conformational polymorphism; *p21*; *p27*; reverse transcription polymerase chain reaction

Control of cell proliferation is managed by a series of checkpoints that regulate cell cycle progression. In recent years, molecular analysis has revealed various abnormalities in the regulatory genes (MacLachlan et al, 1995; Pines, 1995). Retinoblastoma protein (pRb) is a critical target of these cell cycle regulatory genes to promote progression through the $G_1$ phase of the cell cycle.

Sequential activation of cyclin, cyclin-dependent kinase (CDK) complexes is thought to be responsible for orderly transitions through the cell cycle (MacLachlan et al, 1995; Pines, 1995). The abnormal activation of CDK activity leads to underphosphorylation of pRb and may underlie the uncontrolled growth that characterizes neoplasms. The family of low-molecular-weight CDK inhibitors, which includes *p15, p16, p21* and *p27* gene products, is essential in arresting cell cycle progression (MacLachlan et al, 1995; Pines, 1995).

The high incidence of spontaneous pituitary adenoma (almost 100%) that develops in heterozygous *Rb*-knockout mice (Jacks et al, 1992; Hu et al, 1994) implicates the *Rb* pathway in pituitary tumorigenesis, although mutation of the *Rb* gene itself is infrequent in human pituitary adenoma (Cryns et al, 1993; Ikeda et al, 1995). Recently, the homozygous *p27*-knockout mouse was found to develop a high incidence of pituitary adenoma (Fero et al, 1996; Kiyokawa et al, 1996; Nakayama et al, 1996). Both the *Rb* mutation and the $p27^{-/-}$ mice developed morphologically similar tumours originating in the pars intermedia of the pituitary gland. Therefore, the *p27* and *Rb* functions are thought to be in the same regulatory pathway and are important in the tumorigenesis of pituitary adenoma in mice.

The present study investigated genetic changes in *p21* and *p27* in human pituitary adenomas using polymerase chain reaction (PCR)–single-strand conformational polymorphism (SSCP) analysis and direct sequencing.

*Received 29 August 1996*
*Revised 4 April 1997*
*Accepted 15 April 1997*

*Correspondence to:* H Ikeda

### MATERIALS AND METHODS

### Human tissue samples

Twenty-eight samples of pituitary adenoma were obtained at surgery. The histological subtypes were 16 cases of gonadotroph adenoma, five cases of null cell adenoma, four cases of plurihormonal adenoma and three cases of silent corticotroph cell adenoma (Table 1). The resected specimens were frozen immediately in liquid nitrogen and stored at –70°C until analysis.

### DNA isolation

Only pituitary tumour tissues were removed at operation because most of them are soft and easily suckable, in contrast to normal pituitary tissue which is hard to remove by suction or curettage. Adenoma tissues are usually soft because the tissue contains little supportive tissue, such as fibrous stroma and blood vessels; this allowed easy digestion with 1% sodium dodecyl sulphate and proteinase K overnight at 50°C. DNA was extracted with phenol–chloroform, then precipitated with cold ethanol overnight at –20°C. The precipitate was separated by centrifugation. The pellets were dried and resuspended in TE buffer solution (10 mM Tris, 1 mM EDTA; pH 8.0) for storage at 4°C until analysis.

### RNA isolation mRNA was isolated from tissues using the QuickPrepR Micro mRNA purification kit, which combines the disruptive and protective properties of guanidinium thiocyanate with the speed and selectivity of oligo(dT)-cellulose chromatography in a spun-column format (Pharmacia Biotech, Tokyo, Japan).

### Reverse transcription (RT)-PCR analysis of *p21*

Full-length first-strand cDNA was obtained by mixing mRNA template (33 μl) and First-Strand Reaction Mix (T-primed first-strand kit; Pharmacia Biotech). The first-strand cDNA was amplified in a 100 μl mixture containing 6 μl of the first-strand reaction,

**Table 1** Clinicopathological features of 28 patients with pituitary adenoma

| Case no. | Age (years) | Sex | Histology |
|---|---|---|---|
| 1 | 27 | F | Gonadotroph adenoma |
| 2 | 37 | M | Null Cell adenoma |
| 3 | 42 | F | Silent ACTH adenoma |
| 4 | 68 | M | Gonadotroph adenoma |
| 5 | 44 | F | Plurihormonal adenoma |
| 6 | 47 | F | Silent ACTH adenoma |
| 7 | 68 | M | Gonadotroph adenoma |
| 8 | 54 | F | Plurihormonal adenoma |
| 9 | 75 | F | Null cell adenoma |
| 10 | 61 | F | Gonadotroph adenoma |
| 11 | 45 | M | Gonadotroph adenoma |
| 12 | 66 | M | Gonadotroph adenoma |
| 13 | 67 | F | Gonadotroph adenoma |
| 14 | 50 | M | Gonadotroph adenoma |
| 15 | 46 | M | Gonadotroph adenoma |
| 16 | 56 | F | Gonadotroph adenoma |
| 17 | 50 | M | Gonadotroph adenoma |
| 18 | 42 | M | Null cell adenoma |
| 19 | 46 | M | Null cell adenoma |
| 20 | 43 | F | Silent ACTH adenoma |
| 21 | 70 | M | Gonadotroph adenoma |
| 22 | 31 | M | Plurihormonal adenoma |
| 23 | 72 | F | Gonadotroph adenoma |
| 24 | 52 | M | Plurihormonal adenoma |
| 25 | 41 | M | Gonadotroph adenoma |
| 26 | 68 | F | Gonadotroph adenoma |
| 27 | 47 | F | Null cell adenoma |
| 28 | 23 | F | Gonadotroph adenoma |

ACTH, adrenocorticotrophic hormone.

10 $\mu$l of PCR buffer (500 mM potassium chloride, 100 mM Tris, 15 mM magnesium chloride, 0.01% gelatin; pH 8.3), 1 $\mu$l of 20 mM dNTP mixture, 40 pmol of each primer and 2.5 units of *Taq* DNA polymerase (Takara, Ootsu, Japan). The primers used for the amplification of the full length of *p21* were 5′-CACTCAGAGGAGGCGCCATGTCA-3′ and 5′-TTCCAGGACTGCAGGCTTCCT-3′. The control study of the RT-PCR reaction used rabbit globulin (550 bp) obtained by mixing mRNA template and control mixture (Pharmacia Biotech).

**Table 2** Sequence of primers

| Primer | Sequence |
|---|---|
| p21AF | 5′-CACTCAGAGGAGGCGCCATGTCA-3′ |
| p21AR | 5′-TCGAAGTTCCATCGCTCACG-3′ |
| p21BF | 5′-ACTGTGATGCGCTAATGGCG-3′ |
| p21BR | 5′-ATGGTCTTCCTCTGCTGT-3′ |
| p21CF | 5′-ACCTCACCTGCTCTGCTGCA-3′ |
| p21CR | 5′-TTCCAGGACTGCAGGCTTCCT-3′ |
| p27E1AF | 5′-TGCAGACCCGGGAGAAAGATGT-3′ |
| p27E1AR | 5′-ATCGAAATTCCACTTGCGCT-3′ |
| p27E1BF | 5′-AGAGACATGGAAGAGG-3′ |
| p27E1BR | 5′-TGCCATCCTGGCTCTCCT-3′ |
| p27E1CF | 5′-AAGGGCAGCTTGCCCGAGTTCTA-3′ |
| p27E1CR | 5′-GTTGGGAAAGGGCATTACCGT-3′ |
| p27E2F | 5′-TCCCCTGCGCTTAGATTCTTC-3′ |
| p27E2R | 5′-TGATCAACCCACCGAGCTGT-3′ |

### PCR-SSCP analysis

PCR primer pairs for the amplification of the cDNA of *p21* and genomic DNA of *p27* were synthesized by Japan Genetic Research (Sendai, Japan) (Figure 1 and Table 2). PCR fragments were generated from 3 to 6 $\mu$l of the amplified cDNA solution in 100 $\mu$l of a mixture of 200 $\mu$M dTTP, 200 $\mu$M dATP, 200 $\mu$M dGTP, 20 $\mu$M dCTP, 1.0–2.0 mM magnesium chloride, 20 pmol of each primer, 20 mM Tris (pH 8.4 or pH 8.6), 50 mM potassium chloride, 2.5 units of *Taq* DNA polymerase and 0.1 $\mu$Ci of [$\alpha$-$^{32}$P]dCTP. The magnesium chloride concentration and pH of the amplification reaction mixture were optimized for each primer pair. The samples were denatured at 94°C for 5 min. PCR amplification (35–45 cycles) was then carried out using cycles of 1 min 15 s at 94°C (denaturation), 1 min 30 s at 50–55°C (annealing) and 2 min at 71°C (polymerization) using a programmable thermal cycler (PC-800; ASTEC, Fukuoka, Japan). The final polymerization step was performed for 5 min at 71°C. The annealing temperature was optimized for each set of primers. PCR-amplified product solution (5 $\mu$l) was then mixed with 5 $\mu$l of loading solution (95% formamide, 20 mM EDTA, 0.05% xylene cyanol). Diluted samples were denatured at 95°C for 5 min, then loaded on 6% non-denaturing polyacrylamide gels (firstly on a gel containing 10% glycerol and secondly on a gel without glycerol). Electrophoresis

5′ –<br>

GGCGGTCGTGCAGACCCGGGAGAAAGATGTCAAACGTGCGAG TFCTAACGGGAG<br>

CCCTAGCCTGGAGCGGATGGACGCCAGGCAGGCGGATCACCCCAAGCCCTCGGCC<br>

TGCAGGAACCTCTTCGGCCCGGTGGACCACGAAGAGTTAACCCGGGACTTGGAGA<br>

AGCACTGC**AGAGACATGGAAGAGG**CGAGCCAGCGCAAGTFFAATTTCGATTTT<br>

CAGAATCACAAACCCCTAGAGGGCAAGTACGAGTGGAAGAGGTGGAGAAGGGCA<br>

GCTTGCCCGAGTTCTACTACAGACCCCCGCFFCCCCCCAAAGGTGCCTGCAAGGT<br>

GCCGGCGCAGGAGAGCCAGGATGGCAGCGGGAGCCGCCCGGCGGCGCCTTTAATT<br>

GGGGCTCCGGCTAACTCTGAGGACACGCATTTGGTGGACCCAAAGACTGATCCGT<br>

CGGACAGCCAGACGGGGTTAGCGGAGCAATGCGCAGGAATAAGGAAGCGACCTGC<br>

AACCG**ACGgtaatgccctttcccaac**catagaatgtgtttggggccttcaga<br>

cctcacgatacctgatcttactggttgatggcaaattaaaagcttatgggg<br>

–3′

E1A primer pairs: ___<br>

E1B primer pairs: ═══<br>

E1C primer pairs: (shaded)<br>

E1B′ primer pairs: **bold**

**Figure 1** Position of the specific oligonucleotide primers in exon 1 of the *p27* gene

*© Cancer Research Campaign 1997*

**Figure 2** (**A**) PCR-SSCP analysis of the E1A segment of the *p21* gene (non-glycerol gel). (**B**) PCR-SSCP analysis of the E1B segment of the *p21* gene (non-glycerol gel). (**C**) PCR-SSCP analysis of the E1C segment of the *p21* gene (10% glycerol gel). C, control DNA sample; N, no DNA (negative control)

**Figure 3** (**A**) PCR-SSCP analysis of the exon 1-A of the *p27* gene (non-glycerol gel). (**B**) PCR-SSCP analysis of the exon 1-[B+C] of the *p27* gene. Upper panel, 10% glycerol gel; lower panel, non-glycerol gel. Two patterns of migrating bands were detected. The arrows indicate a shifted band. (**C**) PCR-SSCP analysis of the exon 1-C of the *p27* gene (non-glycerol gel). (**D**) PCR-SSCP analysis of the exon E2 of the *p27* gene (10% glycerol gel). C, control DNA sample; N, no DNA (negative control)

© Cancer Research Campaign 1997

was performed both at room temperature and 4°C (cold room) at a constant power of 2–13 W for 6–16 h. Autoradiography was carried out for 5–48 h without an intensifying screen.

### DNA sequencing analysis

The amplified genomic DNA or cDNA fragments used for direct sequencing were purified using Microspin Columns (Pharmacia Biotech). Genomic DNA or cDNA fragments were sequenced in both sense and antisense directions using an ALFred DNA Sequencer (Pharmacia Biotech).

### RESULTS

### SSCP of the *p21* gene

The cDNA of the *p21* gene was divided into segments 1, 2 and 3. No shifted bands were detected in segment 1, 2 or 3 (Figure 2), suggesting that neither point mutations nor polymorphisms were present.

### SSCP of the *p27* gene

The coding region of the *p27* gene consists of two exons (1 and 2). Exon 1 was divided into three segments (1-A, 1-B and 1-C) for detection of mutations by PCR-SSCP. No band shifts were detected in segments exon 1-A, exon 1-C or in exon 2 (Figure 3A, C and D). However, three specimens (case 2, 24 and 26) showed band shifting in exon 1-B and exon 1-[B+C] (Figure 3B). Sequence analysis of these three specimens showed no mutation or polymorphism.

### DISCUSSION

Analysis of 28 specimens of human pituitary tumour found no mutations in the coding region of the *p27* gene. We also found no mutation of the coding region of *p21*. Although p27 protein has homology to the p21 protein and is thought to belong to the products of the same gene family (Polyak et al, 1994; Toyoshima and Hunter, 1994); mice lacking *p21* do not develop pituitary tumours (Deng et al, 1995). An inverse relationship between p16 and pRb inactivation has been found in tumours such as lung cancer and malignant glioma (Hu et al, 1994; Ueki et al, 1996), which suggests a common regulatory mechanism for *p16* and *Rb* functions. Recent *p16* gene analysis found no abnormality in 25 specimens of human pituitary adenoma (Woloschak et al, 1996). Therefore, no abnormalities in the *p16*, *p21* or *p27* genes, which regulate cell proliferation by suppressing hyperphosphorylation and functional inactivation of pRb, are present in human pituitary adenomas.

The embryological morphogenesis of the intermediate lobe of the pituitary gland is unique for each species, but that of the mouse is very similar to that of humans [Ikeda et al, 1988; Ikeda and Yoshimoto, 1991]. Pituitary tumours arising from the intermediate lobe are frequently observed in mice with germline mutation of the *p27* gene. Therefore, a similar gene abnormality may occur in human pituitary tumours. Reduced levels of p16 protein and mRNA have been detected in human pituitary tumours, although no *p16* gene mutation or gene loss was found. This altered expression of the *p16* gene products occurs at a high frequency in human pituitary adenoma (Woloschak et al, 1996) and is due to gene methylation, which interferes with transcription of the gene.

No somatic mutations of the *p21* gene were found in 158 patients with brain tumour (Koopman et al, 1995), and no mutation of this gene was found in 351 DNA specimens from various kinds of malignancies (Shirohara, 1994). Thus, we can speculate that *p21* gene mutations are not involved in the formation of human tumours (Koopman et al, 1995). No mutations of the *p27* gene were found in seven patients with leukaemias (Pietenpol et al, 1995), in more than 20 patients with chronic lymphocytic leukaemia (Bullrich et al, 1995) and in 147 patients with various kinds of malignant solid tumours [Ponce-Castaneda et al, 1995]. However, *p27* acts as a stoichiomeric inhibitor of $G_1$ cyclin-CDKs and even modest changes in the relative levels of *p27* can have a major effect on $G_1$ progression (Kato et al, 1994). According to Hengst and Reed (1996), as variation in the amount of p27 protein occurred, the cell cycle function of p27 was regulated at the level of protein accumulation by post-transcriptional mechanisms, whereas the abundance of the p27 messenger RNA remained unchanged. These findings suggest that further work is required to clarify other mechanisms, such as transcriptional or translational defects, that inactivate the *p27* gene and other genes that cause underphosphorylation of pRb.

#### REFERENCES

Bullrich F, MacLachlan TK, Sang N, Druck T, Veronese ML, Allen SL, Chiorazzi N, Koff A, Heubner H, Croce CM and Giordano A (1995) Chromosomal mapping of members of the cdc2 family of protein kinases, cdk3, cdk6, PISSLRE, and PITALRE, and a cdk inhibitor, p27, to regions involved in human cancer. *Cancer Res* **55**: 1199–1205

Cryns VL, Alexander JM, Klibanski A and Arnold A (1993) The retinoblastoma gene in human pituitary tumors. *J Clin Endocrinol Metab* **77**: 644–646

Deng C, Zhang P, Harper JW, Ellede SJ and Leder P (1995) Mice lacking p21 undergo normal development, but are defective in G1 checkpoint control. *Cell* **82**: 675–684

Fero ML, Rivkin M, Tasch M, Porter P, Carow CE, Firpo E, Polyak K, Tsai LH, Broudy V, Perlmutter RM, Kaushanaky K and Roberts JM (1996) A syndrome of multiorgan hyperplasia with features of gigantism, tumorigenesis, and female sterity in p27-deficient mice. *Cell* **85**: 733–744

Hengst L and Reed SL (1996) Translational control of p27 accumulation during the cell cycle. *Science* **271**: 1861–1864

Hu N, Gustmann A, Herbert DC, Bradley A, Lee WH and Lee EY (1994) Heterozygous Rb-1/+mice are predisposed to tumors of the pituitary gland with a nearly complete penetrance. *Oncogene* **9**: 1021–1027

Ikeda H and Yoshimoto T (1991) The developmental changes in proliferative activity of cells of the murine Rathke's pouch. *Cell Tissue Res* **263**: 41–47

Ikeda H, Suzuki J, Sasano N and Niizuma H (1988) The development and morphogenesis of the human pituitary gland. *Anat Embryol* **178**: 327–336

Ikeda H, Beauchamp RL, Yoshimoto T and Yandell DW (1995) Detection of heterozygous mutation in the retinoblastoma gene in a human pituitary adenoma using PCR-SSCP analysis and direct sequencing. *Endocr Pathol* **6**: 189–196

Jacks T, Frazeli A, Schmitt EM, Bronson RT, Goodell MA and Weinberg RA (1992) Effect of an Rb mutation in the mouse. *Nature* **359**: 295–300

Kato J, Matsuoka M, Polyak K, Massague J and Sherr CJ (1994) Cyclic AMP-induced G1 phase arrest mediated by an inhibitor (p27) of cyclin-dependent kinase 4 activation. *Cell* **79**: 487–496

Kiyokawa H, Kineman RD, Manova-Todorova KO, Soares VC, Hoffman EC, Ono M, Khanam D, Hayday AC, Frohman LA and Koff A (1996) Enhanced growth of mice lacking the cyclin-dependent kinase inhibitor function of p27. *Cell* **85**: 721–732

Koopmann J, Maintz D, Schild S, Schramm J, Louis DN, Wiestler OD and Von Deimling A (1995) Multiple polymorphism, but no mutations, in the WAF1/CIPI gene in human brain tumors. *Br J Cancer* **72**: 1230–1233

MacLachlan TK, Sang N and Giordano A (1995) Cyclin, cyclin-dependent kinases and cdk inhibitors: implication in cell cycle control and cancer. *Critical Reviews in Eukaryotic Gene Expression* **5**: 127–156

© Cancer Research Campaign 1997

Nakayama K, Ishida N, Shirane M, Inomata A, Inoue T, Shishido N, Horii I, Loh DY and Nakayama K (1996) Mice lacking p27 display increased body size, multiple organ hyperplasia, retinal dysplasia, and pituitary tumors. *Cell* **85**: 707–720

Pietenpol JA, Bohlander SK, Sato Y, Papadopoulos N, Liu B, Friedman C, Trask BJ, Roberts JM, Kinzler KW, Rowley JD and Vogelstein B (1995) Assignment of the human p27 gene to 12p13 and its analysis in leukemias. *Cancer Res* **55**: 1206–1210

Pines J (1995) Cyclins, CDKs and cancer. *Semin Cancer Biol* **6**: 63–72

Ponce-Castaneda MV, Lee MH, Latres E, Polyak K, Lacombe L, Montgomery K, Mathew S, Krauter K, Sheinfeld J, Massague J and Cordon-Cardo C (1995) p27: chromosomal mapping to 12p12–12p13.1 and absence of mutation in human tumors. *Cancer Res* **55**: 1211–1214

Polyak K, Lee MH, Erdjument-Bromage H, Koff A, Roberts JM, Tempst P and Massague J (1994) Cloning of p27, a cyclin-dependent kinase inhibitor and a potential mediator of extracellular antimitogenic signals. *Cell* **78**: 59–66

Shiohara M, Ei-Deiry WS, Wada M, Nakamaki T, Takeuchi S, Yang R, Chen DL, Vogelstein B and Koeffler HP (1994) Absence of WAF1 mutations in a variety of human malignancies. *Blood* **84**: 3781–3784

Toyoshima H and Hunter T (1994) p27, a novel inhibitor of G1 cyclin-cdk protein kinase activity, is related to p21. *Cell* **78**: 67–74

Ueki K, Ono Y, Henson JW, Efird JT, Von Deimling A and Louis DN (1996) CDKN2/p16 or RB alteration occur in the majority of glioblastoma and are inversely correlated. *Cancer Res* **56**: 150–153

Woloschak M, Yu A, Xiao J and Post KD (1996) Frequent loss of the p16 gene product in human pituitary tumors. *Cancer Res* **56**: 2493–2496

© Cancer Research Campaign 1997


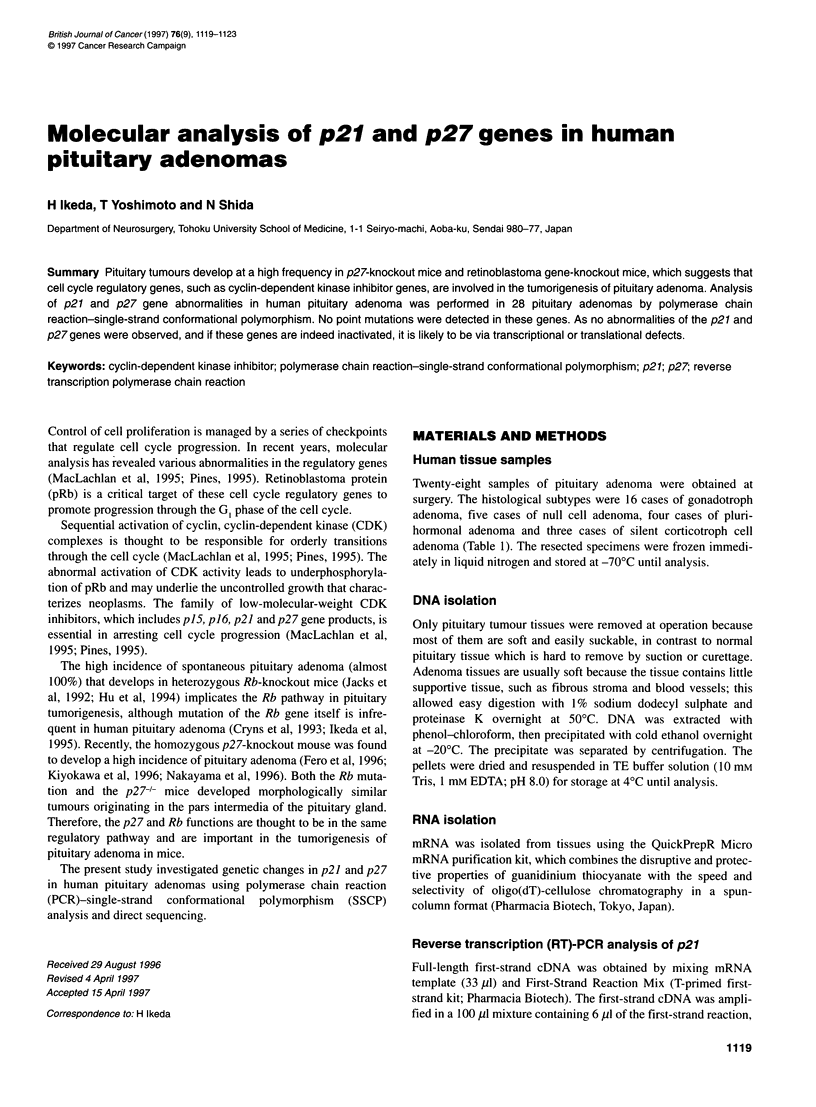

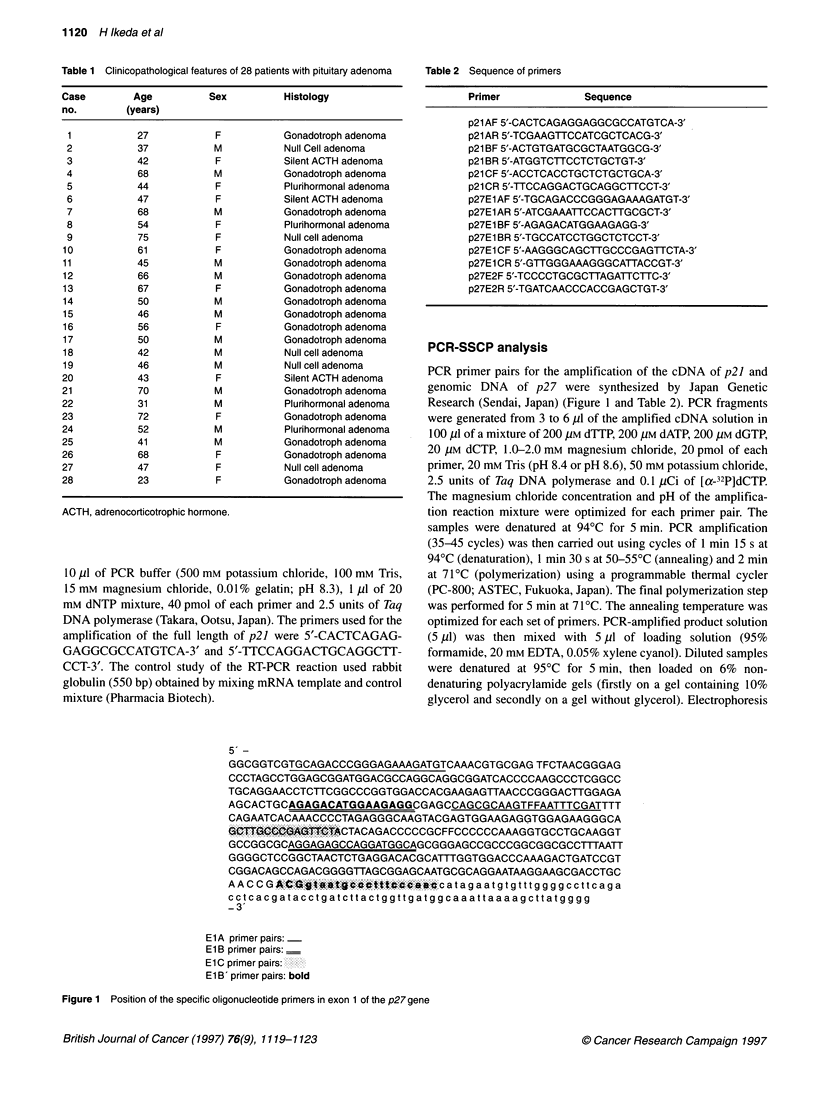

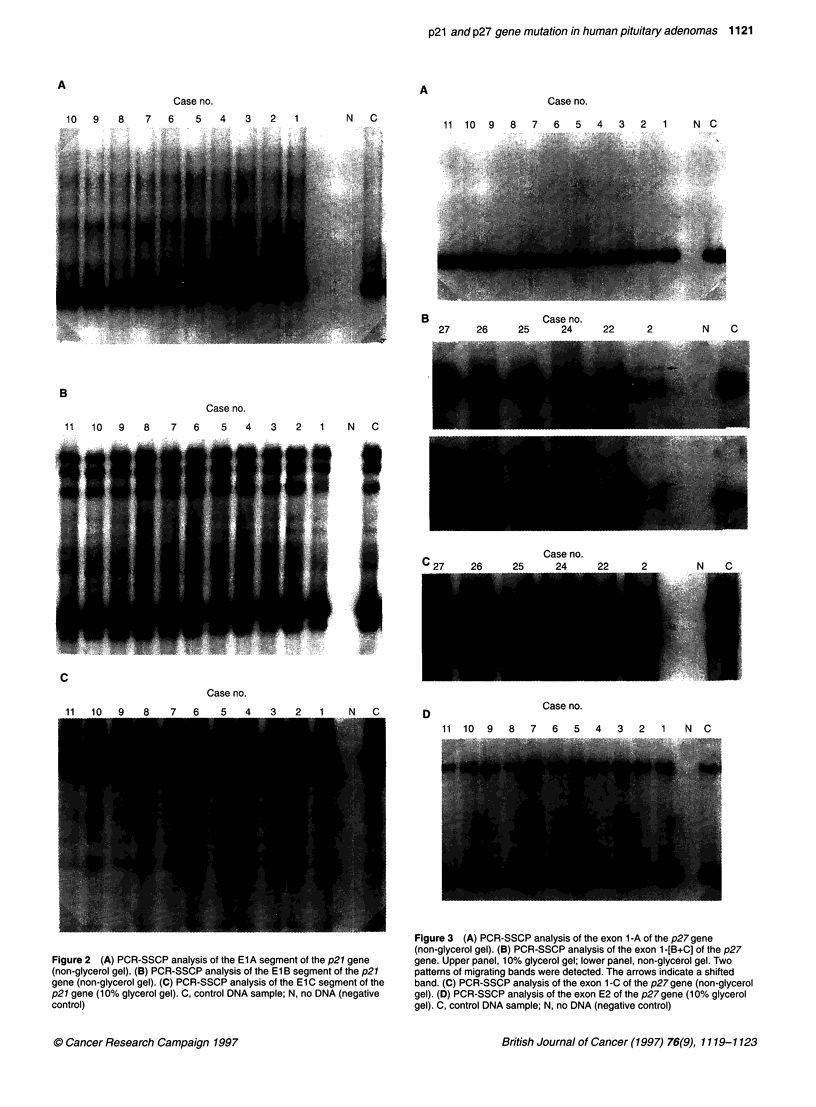

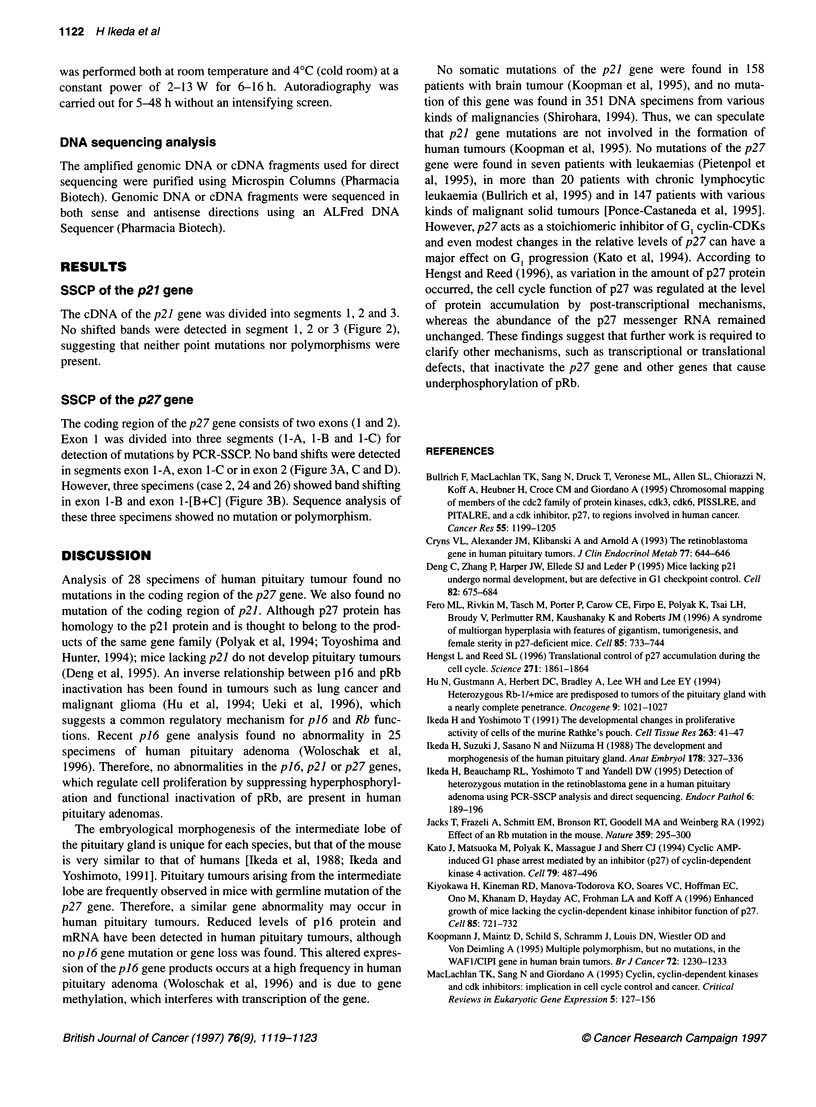

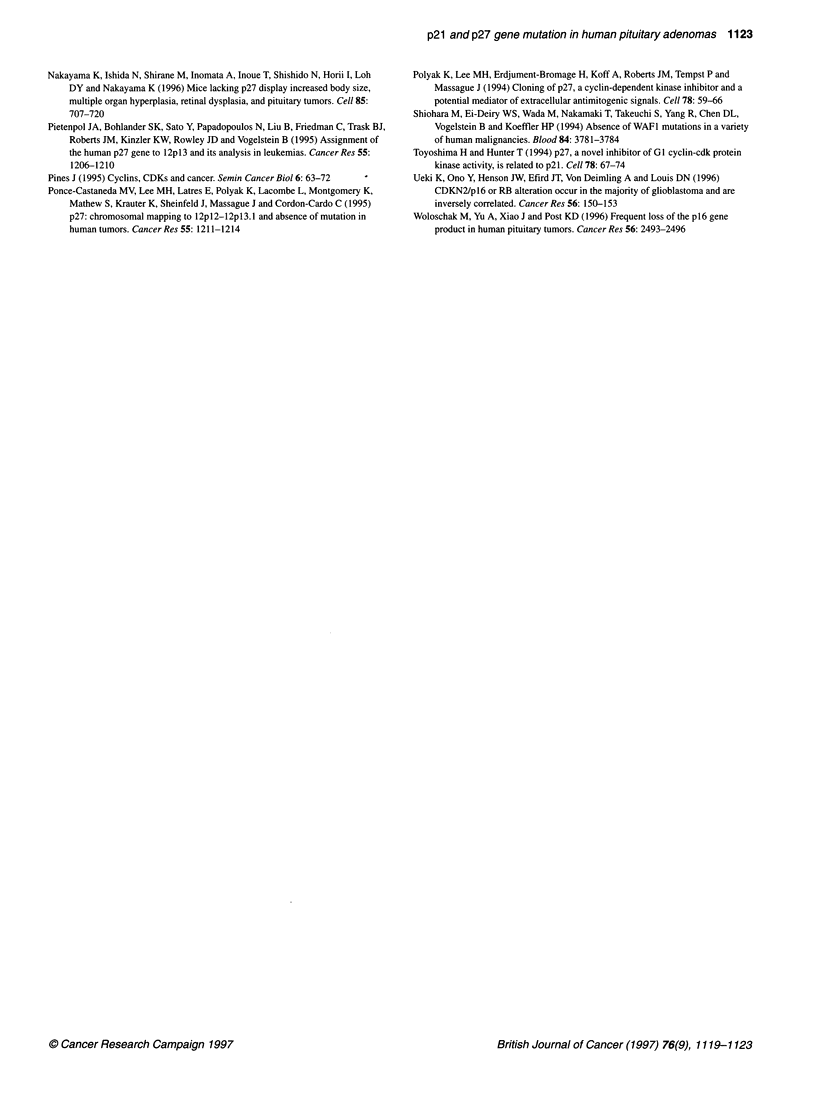


## References

[OCR_00386] Bullrich F., MacLachlan T. K., Sang N., Druck T., Veronese M. L., Allen S. L., Chiorazzi N., Koff A., Heubner K., Croce C. M. (1995). Chromosomal mapping of members of the cdc2 family of protein kinases, cdk3, cdk6, PISSLRE, and PITALRE, and a cdk inhibitor, p27Kip1, to regions involved in human cancer.. Cancer Res.

[OCR_00393] Cryns V. L., Alexander J. M., Klibanski A., Arnold A. (1993). The retinoblastoma gene in human pituitary tumors.. J Clin Endocrinol Metab.

[OCR_00397] Deng C., Zhang P., Harper J. W., Elledge S. J., Leder P. (1995). Mice lacking p21CIP1/WAF1 undergo normal development, but are defective in G1 checkpoint control.. Cell.

[OCR_00402] Fero M. L., Rivkin M., Tasch M., Porter P., Carow C. E., Firpo E., Polyak K., Tsai L. H., Broudy V., Perlmutter R. M. (1996). A syndrome of multiorgan hyperplasia with features of gigantism, tumorigenesis, and female sterility in p27(Kip1)-deficient mice.. Cell.

[OCR_00408] Hengst L., Reed S. I. (1996). Translational control of p27Kip1 accumulation during the cell cycle.. Science.

[OCR_00412] Hu N., Gutsmann A., Herbert D. C., Bradley A., Lee W. H., Lee E. Y. (1994). Heterozygous Rb-1 delta 20/+mice are predisposed to tumors of the pituitary gland with a nearly complete penetrance.. Oncogene.

[OCR_00420] Ikeda H., Suzuki J., Sasano N., Niizuma H. (1988). The development and morphogenesis of the human pituitary gland.. Anat Embryol (Berl).

[OCR_00417] Ikeda H., Yoshimoto T. (1991). Developmental changes in proliferative activity of cells of the murine Rathke's pouch.. Cell Tissue Res.

[OCR_00423] Ikeda Hidetoshi, Beauchamp Roberta L., Yoshimoto Takashi, Yandell David W. (1995). Detection of Heterozygous Mutation in the Retinoblastoma Gene in a Human Pituitary Adenoma Using PCR-SSCP Analysis and Direct Sequencing.. Endocr Pathol.

[OCR_00430] Jacks T., Fazeli A., Schmitt E. M., Bronson R. T., Goodell M. A., Weinberg R. A. (1992). Effects of an Rb mutation in the mouse.. Nature.

[OCR_00434] Kato J. Y., Matsuoka M., Polyak K., Massagué J., Sherr C. J. (1994). Cyclic AMP-induced G1 phase arrest mediated by an inhibitor (p27Kip1) of cyclin-dependent kinase 4 activation.. Cell.

[OCR_00439] Kiyokawa H., Kineman R. D., Manova-Todorova K. O., Soares V. C., Hoffman E. S., Ono M., Khanam D., Hayday A. C., Frohman L. A., Koff A. (1996). Enhanced growth of mice lacking the cyclin-dependent kinase inhibitor function of p27(Kip1).. Cell.

[OCR_00446] Koopmann J., Maintz D., Schild S., Schramm J., Louis D. N., Wiestler O. D., von Deimling A. (1995). Multiple polymorphisms, but no mutations, in the WAF1/CIP1 gene in human brain tumours.. Br J Cancer.

[OCR_00451] MacLachlan T. K., Sang N., Giordano A. (1995). Cyclins, cyclin-dependent kinases and cdk inhibitors: implications in cell cycle control and cancer.. Crit Rev Eukaryot Gene Expr.

[OCR_00460] Nakayama K., Ishida N., Shirane M., Inomata A., Inoue T., Shishido N., Horii I., Loh D. Y., Nakayama K. (1996). Mice lacking p27(Kip1) display increased body size, multiple organ hyperplasia, retinal dysplasia, and pituitary tumors.. Cell.

[OCR_00466] Pietenpol J. A., Bohlander S. K., Sato Y., Papadopoulos N., Liu B., Friedman C., Trask B. J., Roberts J. M., Kinzler K. W., Rowley J. D. (1995). Assignment of the human p27Kip1 gene to 12p13 and its analysis in leukemias.. Cancer Res.

[OCR_00472] Pines J. (1995). Cyclins, CDKs and cancer.. Semin Cancer Biol.

[OCR_00480] Polyak K., Lee M. H., Erdjument-Bromage H., Koff A., Roberts J. M., Tempst P., Massagué J. (1994). Cloning of p27Kip1, a cyclin-dependent kinase inhibitor and a potential mediator of extracellular antimitogenic signals.. Cell.

[OCR_00474] Ponce-Castañeda M. V., Lee M. H., Latres E., Polyak K., Lacombe L., Montgomery K., Mathew S., Krauter K., Sheinfeld J., Massague J. (1995). p27Kip1: chromosomal mapping to 12p12-12p13.1 and absence of mutations in human tumors.. Cancer Res.

[OCR_00485] Shiohara M., el-Deiry W. S., Wada M., Nakamaki T., Takeuchi S., Yang R., Chen D. L., Vogelstein B., Koeffler H. P. (1994). Absence of WAF1 mutations in a variety of human malignancies.. Blood.

[OCR_00490] Toyoshima H., Hunter T. (1994). p27, a novel inhibitor of G1 cyclin-Cdk protein kinase activity, is related to p21.. Cell.

[OCR_00494] Ueki K., Ono Y., Henson J. W., Efird J. T., von Deimling A., Louis D. N. (1996). CDKN2/p16 or RB alterations occur in the majority of glioblastomas and are inversely correlated.. Cancer Res.

[OCR_00499] Woloschak M., Yu A., Xiao J., Post K. D. (1996). Frequent loss of the P16INK4a gene product in human pituitary tumors.. Cancer Res.

